# Anti-Cancer Effects of Lycopene in Animal Models of Hepatocellular Carcinoma: A Systematic Review and Meta-Analysis

**DOI:** 10.3389/fphar.2020.01306

**Published:** 2020-08-21

**Authors:** Abraham Nigussie Mekuria, Abera Kenay Tura, Bisrat Hagos, Mekonnen Sisay, Jemal Abdela, Kirubel Minsamo Mishore, Birhanu Motbaynor

**Affiliations:** ^1^Department of Pharmacology, School of Pharmacy, College of Health and Medical Sciences, Haramaya University, Harar, Ethiopia; ^2^School of Nursing and Midwifery, College of Health and Medical Sciences, Haramaya University, Harar, Ethiopia; ^3^Social Pharmacy Unit, School of Pharmacy, College of Health and Medical Sciences, Haramaya University, Harar, Ethiopia; ^4^Department of Clinical Pharmacy, School of Pharmacy, College of Health and Medical Sciences, Haramaya University, Harar, Ethiopia; ^5^Department of Pharmaceutical Chemistry, School of Pharmacy, College of Health and Medical Sciences, Haramaya University, Harar, Ethiopia

**Keywords:** lycopene, carotenoids, hepatocellular carcinoma, liver cancer, animal studies, meta-analysis

## Abstract

**Introduction:**

Globally, hepatocellular carcinoma (HCC) is the sixth most diagnosed cancer and the third important cause of cancer-related death. As there are only two targeted drugs for the treatment of advanced HCC—that merely extend survival by a few months—the need for alternative treatments is inevitable. Lycopene, a carotenoid that is known to be most abundant in red tomatoes and tomato-based products, has been investigated for its anticancer activity in various types of cancers including HCC. This review was conducted to evaluate the effects of lycopene on HCC from animal models to pave the way for further clinical studies.

**Methods:**

Electronic databases and search engines including PubMed, EMBASE, and Google Scholar were searched for original records addressing the anticancer effect of lycopene in animal models of HCC. Data were extracted using a format prepared in Microsoft Excel and exported to Stata 15.0 for analyses. A meta-analysis was performed using a random-effects model at a 95% confidence level for the outcome measures: tumor incidence, number, and growth (tumor volume and size). The presence of publication bias between studies was evaluated using Egger’s test and funnel plot. The study protocol was registered in the PROSPERO database with reference number: CRD42019159312.

**Results:**

The initial database search yields 286 articles, of which 15 studies met the inclusion criteria. The characteristics of the included studies were a bit diversified. The studies involved a total of 644 animals (312 treatment and 332 control groups) and mice shared the majority (488) followed by rats (117) and ferrets (39). The meta-analysis showed that lycopene significantly reduced the incidence [RR 0.8; 95% CI 0.69, 0.92 (*p*=0.00); I^2^ = 30.4%, *p*=0.16; n=11], number [SMD-1.83; 95% CI -3.10, -0.57 (*p*=0.01); I^2^ = 95.9%, *p*=0.00; n=9], and growth [SMD -2.13; 95% CI -4.20, -0.04 (*p*=0.04); I^2^ = 94.6%, *p*=0.00; n=4] of HCC.

**Conclusions:**

Administration of* *lycopene appears to inhibit the initiation and progression of cancer in animal models of HCC. However, more controlled and thorough preclinical studies are needed to further evaluate its anti-HCC effects and associated molecular mechanisms.

## Introduction

Hepatocellular carcinoma (HCC) is a common type of primary liver cancer that has become a major public health problem ranked as the sixth most common cancer and the third leading cause of cancer-related deaths (Bray et al., 2018). Surgical resection, liver transplantation, and locoregional therapies are potential curative treatment options for early or intermediate stage HCC (Kirstein and Wirth, 2020). For patients with advanced HCC, however, only two targeted drugs—multi-kinase inhibitor sorafenib, and the vascular endothelial growth factor-2 (VEGFR-2) antagonist ramucirumab—that only increase patient survival by few months exist (Lurje et al., 2019; Vogel and Saborowski, 2020). Therefore, the development of alternative methods of treatment needs urgent attention.

Lycopene, a bioactive deep-red pigment naturally synthesized by fruits and vegetables, is the most abundant carotenoid in red tomatoes and tomato-based products, including ketchup, tomato juice, and pizza sauce (Ilahy et al., 2019). Human and experimental studies have reported that lycopene can influence the development and progression of HCC (Mein et al., 2008; Ip and Wang, 2013; Stice et al., 2018; Thomas et al., 2020). For instance, in studies conducted using hepatoma cell lines lycopene inhibited cell growth, migration, and invasion (Huang et al., 2008; Yang et al., 2012; Jhou et al., 2017). Besides, orally administered lycopene attenuates liver-specific carcinogen diethylnitrosamine (DEN)-induced hepatocarcinogenesis in rodents (Ip et al., 2014; Sahin et al., 2014; Bhatia et al., 2018). To our knowledge, no published systematic review or meta-analysis that reported evidence vis-à-vis the effects of lycopene in animal models of HCC. Hence, we conducted a systematic review and meta-analysis to evaluate the anti-HCC effects of lycopene in animal models of HCC.

## Materials and Methods

### Study Protocol

This study was conducted following the Preferred Reporting Items for Systematic Reviews and Meta-Analyses (PRISMA) guidelines (Moher et al., 2009). The completed checklist is provided as supplementary material (**Additional file 1: Table S1**). The study protocol is registered on PROSPERO with reference number CRD42019159312 and available at: https://www.crd.york.ac.uk/prospero/#recordDetails

### Data Sources and Search Strategy

We conducted an electronic literature search in PubMed and EMBASE from onset to February 29^th^, 2020 using the following keywords and indexing terms: “lycopene”, “carotenoids”, “hepatocellular carcinoma”, and “animal model”. Reference lists of identified citations and Google Scholar were also searched to identify additional studies. Boolean operators (AND, OR) or its meta and truncation were used when appropriate to increase the number of relevant articles. An example of the search strategy employed in PubMed is provided as supplementary material (**Additional file 2: Table S2**).

### Study Selection and Inclusion Criteria

Two reviewers independently reviewed the articles for inclusion through scanning of titles and abstracts for relevance. Articles deemed important or for which decisions were difficult to exclude were retained for full-text review. Similarly, two reviewers independently reviewed the articles against pre-defined inclusion criteria: used animal models of HCC, reported anticancer effects directly generated by lycopene treatment, and controlled studies with a separate control group, published in English. There was no restriction based on the year of publication, or sample size. Disagreement during the full-text review was resolved through discussion until unanimity.

### Data Extraction and Quality Assessment

By using a format prepared in Microsoft Excel the following items were extracted: author, year, characteristics the animal models (species/strain, age, sex, weight, and type), number of animals in the experimental and control group, the dose of lycopene, timing, duration and route of administration, and outcomes measures including tumor size (TS), tumor volume (TV), tumor number, tumor incidence (TI), and molecular markers.

The quality of the included studies was evaluated by using the Collaborative Approach to Meta-Analysis and Review of Animal Data from Experimental Studies (CAMARADES) checklist (Macleod et al., 2004). The items consisted of (1) publication in a peer-reviewed journal; (2) statement of control of temperature; (3) randomization to treatment or control; (4) allocation concealment; (5) blinded assessment of outcome; (6) use of a suitable animal model of HCC; (7) sample size calculation; (8) avoidance of anesthetics with marked intrinsic properties; (9) statement of compliance with regulatory requirements; and (10) statement of potential conflicts of interest. A point was given for each criterion reported. The potential score ranges from 0 to 10, with higher scores indicating greater methodological rigor. Two independent investigators performed a quality assessment and disagreements were resolved through discussion by reviewing the full text together.

### Data Processing and Statistical Analysis

The extracted data were analyzed using Stata 15.0. Risk ratio (RR) was used as the effect measure for the outcome of tumor incidence and the standardized mean difference (SMD) for the outcome measures of tumor growth (TV, TS) and number. Considering the variation in true effect sizes across the population, the random-effects model was applied for the analysis at a 95% confidence level. The significance of heterogeneity of the studies was assessed using I^2^ statistics based on Cochran’s *Q* test, *I^2^* returns, and the percent variation across studies. Subgroup analysis was performed based on type of HCC model, species/strain, and lycopene dose. The presence of publication bias was evaluated using Egger’s regression (Egger et al., 1997) tests and presented with funnel plots. A statistical test with a *p*-value of less than 0.05 was deemed to be significant.

## Results

### Study Selection

**Figure 1:**
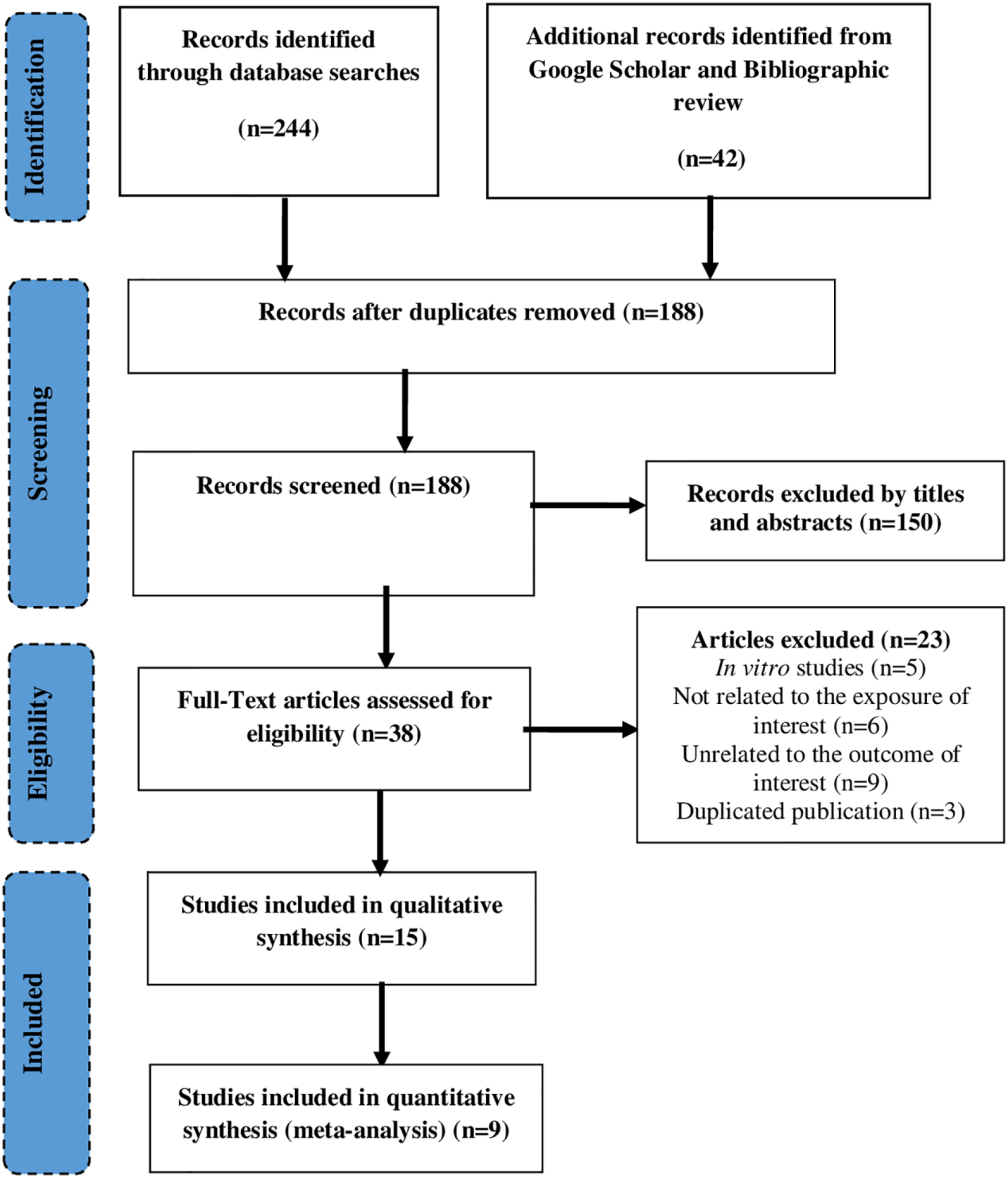
PRISMA flow chart describing the selection process.

A total of 286 studies were identified through the database search (n=244), and Google scholar or bibliographic review (n=42). After removing duplicates (n=98), 188 records were screened using titles and abstracts of which 38 studies retained for the full-text review. Finally, a total of 15 studies were included in the systematic review (Watanabe et al., 2001; Toledo et al., 2003; Takahashi et al., 2010; Wang et al., 2010; Gupta et al., 2013a; Gupta et al., 2013b; Gupta et al., 2013c; Ip et al., 2013; Ip et al., 2014; Sahin et al., 2014; Bhatia et al., 2015; Stice et al., 2015; Aizawa et al., 2016; Gupta et al., 2016; Bhatia et al., 2018), of which 10 studies (Watanabe et al., 2001; Toledo et al., 2003; Takahashi et al., 2010; Wang et al., 2010; Gupta et al., 2013c; Ip et al., 2013; Ip et al., 2014; Sahin et al., 2014; Stice et al., 2015; Aizawa et al., 2016) included in the meta-analysis. The major reasons for exclusion were not containing the outcome of interest (n=9), not related to exposure of interest, and being *in vitro* study (n=5) (**Figure 1**).

### Study Characteristics

The studies were published between 2001 and 2018 and employed different animals. More than 1/3 of the studies used BALB/c mice (Gupta et al., 2013a; Gupta et al., 2013b; Gupta et al., 2013c; Bhatia et al., 2015; Gupta et al., 2016; Bhatia et al., 2018) and others used Wistar rats (Toledo et al., 2003; Sahin et al., 2014; Stice et al., 2015), Sprague-Dawley rats (Wang et al., 2010), WT/BCO2-KO mice (Ip et al., 2014), C3H/HeN mice (Takahashi et al., 2010), C57BL/6J mice (Ip et al., 2013), ferrets (Aizawa et al., 2016), and Long-Evans Cinnamon rats (Watanabe et al., 2001). Six of the 15 studies enrolled female animals (Gupta et al., 2013a; Gupta et al., 2013b; Gupta et al., 2013c; Bhatia et al., 2015; Gupta et al., 2016; Bhatia et al., 2018). Although more than half of the studies used 5 mg/kg dose and given before administration of the carcinogen, there was significant variation among studies in terms of dose, frequency, and duration of lycopene administration. Six of the 15 studies have only reported the expression of inflammatory (Stice et al., 2015; Bhatia et al., 2018), redox (Gupta et al., 2013a; Gupta et al., 2013b; Bhatia et al., 2018), proliferation, and apoptosis (Bhatia et al., 2015; Gupta et al., 2016) markers as their main outcome measures in an effort to elucidate the anti-HCC mechanism of lycopene (**Table 1**).

**Table 1:** Characteristics of the included studies.

1^st^ author & year	Animal model characteristics					Experimental group (n)	Control group(n)	Lycopene administration		Outcome measures
	Strain/Species	Gender	Age	Weight	HCC Model			Timing of administration	Dosage regimen	
Watanabe et al., 2001	LEC rats	M	6wk	NR	LEC	HCC+Ly (20)	LEC rats (20)	NA	5 mg/kg, daily for 70 wks *via* diet	TV
Toledo et al., 2003	Wistar rats	M	NR	40-45g	DEN+AAF	Ly + DEN+AAF (8)	Corn oil+ DEN+AAF (8)	2wks before DEN & 4wks before AAF	5 mg/kg, 3x a wk for 8 wks *via* gavage	TI, TS, TN
Takahashi et al., 2010	C3H/HeN mice with XPA+/+, +/-, & -/-	M	6wk	NR	C3H/HeN	HCC+Ly (14, 15, 17)	C3H/HeN mice (16, 18, 18)	NA	5 mg/kg, daily for 66wks *via* diet	TI, TS, TN, 8OHdG
Wang et al., 2010	Sprague-Dawley rats	M	8wk	NR	HFD	HFD+Ly (8)	HFD (8)	3 days after DEN	15 mg/kg, daily for 6 wks *via* diet	TI, JNK, ERK1/2, oxidative stress & TNF-α, IL-1β, IL-12, Nrf2, NF-κB p65, HO-1, MDA, 4-HNE
Gupta et al., 2013a	Balb/c mice	F	NR	25-30g	DEN	Ly +DEN (5)	DEN (5)	2wks before DEN	5 mg/kg, 3x a wk for 24 wks *via* diet	bcl-2, caspase 3, caspase 9, p53, LPO, Glutathione redox ratio
Gupta et al., 2013b	Balb/c mice	F	NR	25-30g	DEN	Ly +DEN (10)	DEN (10)	2wks before DEN	5 mg/kg, 3x a wk for 12 wks *via* diet	Chromosomal analysis, Cytochrome P450 m LPO, GSH, GST, *GSH peroxidase*, GR, CAT, SOD, LFTs
Gupta et al., 2013c	Balb/c mice	F	NR	25-30g	DEN	Ly +DEN (20)	DEN (16)	2wks before DEN	5 mg/kg, 3x a wk for 24 wks *via* diet	TI, TN
Ip et al., 2013	C57Bl/6J mice	M	2wk	NR	DEN/HFD	HFD+APO10LA* (24)	HFD (24)	4wks after DEN	10 mg/kg, 2-3 x a wk for 24 wks *via* diet	TN, TV, SIRT1. NF-κB p65, FoxO1, AMPKα, STAT3, TNFα, IL-6, caspase-1 cyclin D1.
Ip et al., 2014	WT & BCO2-KO mice	M	2wk	NR	DEN/HFD	HFD+Ly (26, 80)	HFD (26, 100)	4 wks after DEN	10 mg/kg, 2-3 x a wk for 24 wks *via* diet	TI, TN, ER stress, mTOR, inflammatory markers, met
Sahin et al., 2014	Wistar rats	M	8wk	180–200g	DEN	DEN+Ly (12)	DEN (9)	4wks before DEN	20 mg/kg, 3x a wk for 24 wks *via* diet	TI, TN, TV, NF-*κ*B p65, COX-2, Nrf2, HO-1, mTOR, 70S6K1, 4EBP1 & Akt
Stice et al., 2015	Wistar rats	M	8wk	NR	DEN/Ethanol	Ethanol+Ly(12)	Ethanol (12)	8 wks after DEN & 2wks after ethanol	1.1 mg/kg, daily for 4 wks *via* diet	ER stress, CYP2E1, inflammatory markers
Bhatia et al., 2015	Balb/c mice	F	NR	25-30g	DEN	Ly +DEN (10)	DEN (10)	2wks before DEN	5 mg/kg, 3x a wk for 12 wks *via* diet	LFTs, AFP, HIF-1a, VEGF, CD31, MMP-2, & MMP-9
Aizawa et al., 2016	Ferret	M	NR	1.1-1.7kg	NNK	NNK+ Ly (10, 9)	NNK + placebo (10, 10)	3wks before NNK	2.2 mg & 6.6mg/kg, daily for 26 wks	TI, CYP2E1, cyclin D1, MMP-2, NF-κB
Gupta et al., 2016	Balb/c mice	F		25-30g	DEN	Ly +DEN (7)	DEN (7)	2wks before DEN	5 mg/kg, 3x a wk for 24 wks *via* diet	PCNA, Cyclin D1, p21, glycolytic enzymes activity
Bhatia et al., 2018	Balb/c mice	F	NR	25-30g	DEN	Ly +DEN (5)	DEN (5)	2wks before DEN	5 mg/kg, 3x a wk for 12 wks *via* diet	LFTs, inflammatory markers, LPO, GSH, GST, *GSH peroxidase*, GR, CAT, SOD

*lycopene metabolite. 2-AAF, 2-acetylaminofluorene; 4-HNE, 4-hydroxynonenal; 8OHdG; 8-hydroxy-2’-deoxyguanosine; AFP, alpha feto protein; AMPK, AMP-activated protein kinase; APO10LA, Apo-10’-lycopenoic acid; BCO2-KO, beta-carotene-9’,10’-oxygenase-knockout; CAT, catalase; CD31, cluster of differentiation 31; COX-2, cyclooxygenase-2; CYP, Cytochrome P450; DEN, Diethylnitrosamine; ER, endoplasmic reticulum; ERK1/2, extracellular signal-regulated kinase; GSH, glutathione; GST, glutathione-s transferase; GR, glutathione reductase; HFD, high-fat diet; HIF, hypoxia induced factor; HO-1, Heme oxygenase-1; IL, interleukin; JNK, c-Jun N-terminal kinase; LEC, Long-Evans Cinnamon; LFTs, liver function tests; LPO, lipid peroxidation; Ly, lycopene; MDA, malondialdehyde; MMPs, Matrix metalloproteinases; NA, not applicable; mTOR, mammalian target of rapamycin; NF-κB, nuclear factor- κB; NNK, 4-(N-methyl-N-nitrosamino)-1-(3-pyridyl)-1-butanone;NR, not reported; Nrf2, NF-E2-related factor 2; PCNA, proliferating cell nuclear antigen; SOD, superoxide dismutase; STAT3, signal transducer & activator of transcription 3; TN, tumor number; TNF-α, tumor necrosis factor-α; TS, tumor size; TI, tumor incidence; TV, Tumor volume; VEGF, vascular endothelial growth factor; WT, wild-type; XPA, xeroderma pigmentosum complementation group A.

The median quality score checklist items scored was 6 out of 10 (interquartile range, 5–7; **Additional file 3: Table S3**). All the included studies were peer-reviewed publications, used a suitable animal model of HCC, and avoided the use of anesthetics with marked intrinsic properties. However, none of the studies reported the detailed protocol regarding the conducted experiment, and all of the studies did not report sample size calculations except one study (Sahin et al., 2014).

### Meta-Analysis

#### Tumor Incidence

**Figure 2:**
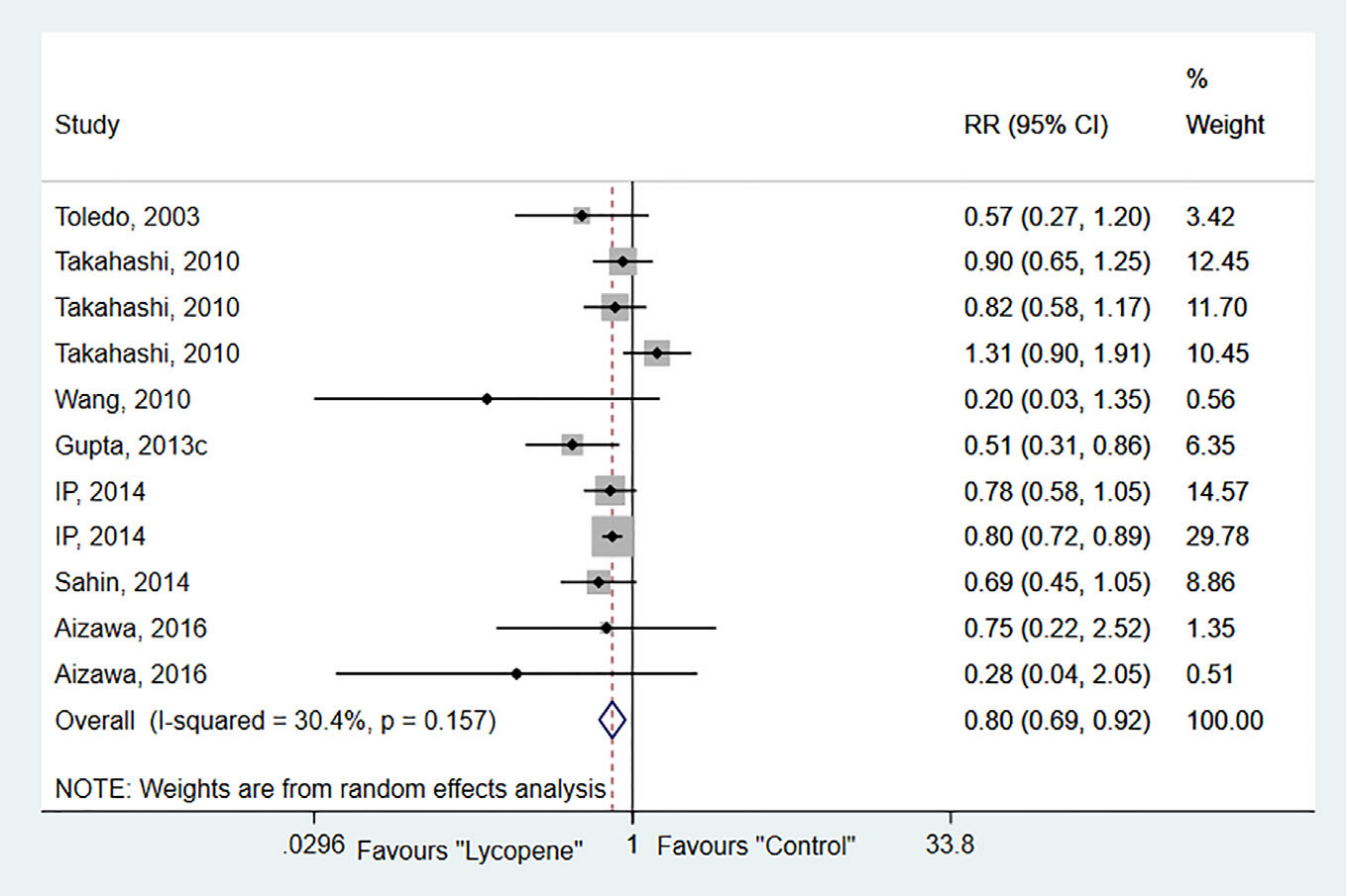
Forest plot of the effects of lycopene on tumor incidence.

Seven studies with 11 independent experiments were included to investigate the effect of lycopene treatment on HCC incidence. The pooled estimate indicated a significant decrease in tumor incidence (RR 0.8; 95% CI 0.69, 0.92 (*p*=0.00), and between-study heterogeneity was not evident (*I^2 =^* 30.4%, *p*=0.16; **Figure 2**). Subgroup analysis indicated a significant reduction of tumor incidence in rats [RR 0.63; 95% CI 0.44, 0.90 (*p*=0.01); *I^2^* = 0.0%, *p*=0.44]; chemically-induced HCC model [RR 0.60; 95% CI 0.45, 0.80 (*p*=0.00); *I^2^* = 0.00%, *p*=0.83]; at a dose of ≥5mg/kg [RR 0.75; 95% CI 0.64, 0.87 (*p*=0.00); *I^2^* = 16.3%, *p*=0.31]; and duration of lycopene therapy ≤ 24 weeks with no evidence of heterogeneity (**Table 2**).

**Table 2:** Subgroup analyses of the effects of lycopene on tumor incidence and number.

Subgroup	Experiments, N	RR/SMD (95% CI)	*p*-value	Tests for heterogeneity		
				Q	*p*	I^2^
**Tumor incidence**						
All studies	11	0.80 (0.69, 0.92)	0.00	14.4	0.16	30.4
Strain/species						
Rats	3	0.63 (0.44, 0.90)	0.01	1.6	0.44	0.0%
Mice	6	0.84 (0.71,0.99)	0.04	9.7	0.08	48.5%
Ferrets	2	0.57 (0.20,1.62)	0.29	0.7	0.41	0.0%
HCC model						
Spontaneous	3	0.98 (0.75, 1.28)	0.89	3.5	0.17	43.5%
Chemical	5	0.60 (0.45,0.80)	0.00	1.5	0.83	0.0%
Diet	3	0.79 (0.71,0.89)	0.00	2.0	0.36	1.4%
Lycopene dose						
<5mg/kg	5	0.92 (0.73,1.17)	0.51	5.5	0.24	27.5%
≥5mg/kg	6	0.75 (0.64,0.87)	0.00	6.0	0.31	16.3%
Duration						
<24 weeks	6	0.75 (0.66, 0.86)	0.00	5.6	0.35	10.8%
>24 weeks	5	0.95 (0.74, 1.22)	0.69	5.2	0.27	23.0%
**Tumor number**						
All studies	9	-1.83 (-3.10, -0.57)	0.01	195.6	0.00	95.9%
Strain/species						
Rats	2	-2.46 (-3.34, -1.58)	0.00	0.5	0.49	0.0%
Mice	7	-1.65 (-3.15, -0.15)	0.03	192.4	0.00	96.9%
HCC model						
Spontaneous	3	0.15 (-0.25, 0.56)	0.46	2.0	0.36	1.1%
Chemical	3	-1.84 (-3.16, -0.52)	0.01	9.4	0.01	78.7%
Diet	3	-3.7 (-4.42, -2.98)	0.00	5.2	0.07	61.6%
Lycopene dose						
<5mg/kg	4	-0.42 (-1.41,0.57)	0.41	17.6	0.00	82.9%
≥5mg/kg	5	-2.85 (-4.18, -1.52)	0.00	59.1	0.00	93.2%
Duration						
<24 weeks	6	-2.85 (-4.02, -1.68)	0.00	59.1	0.00	91.5%
>24 weeks	3	0.154(-0.25, 0.56)	0.46	2.0	0.36	1.1%

CI, confidence interval; RR, Risk Ratio; SMD, standardized mean difference.

#### Tumor Number

**Figure 3:**
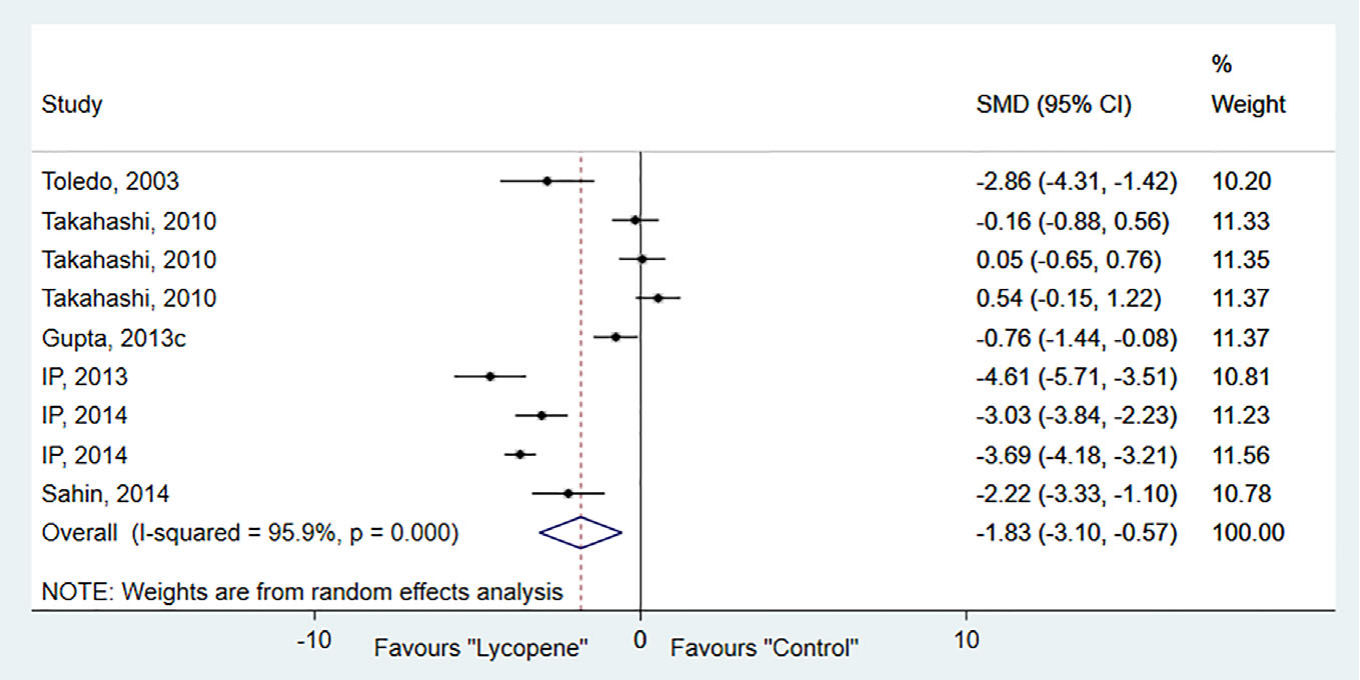
Forest plot of the effects of lycopene on tumor number.

Six studies, containing 9 independent experiments, were included to investigate the effect of lycopene on tumor numbers. The overall pooled analysis showed a significant reduction in tumor number (SMD-1.83; 95% CI -3.10, -0.57; *p*=0.01). However, the test for heterogeneity was significant (I^2^ = 95.9%, *p*=0.00; **Figure 3**). Subgroup analysis showed a significant decrease in tumor number in rats than mice and ferrets, with no evidence of heterogeneity [SMD -2.46; 95% CI -3.34, -1.58 (*p*=0.00); I^2^ = 0.0%, *p*=0.49] (**Table 2**).

#### Tumor Growth

**Figure 4:**
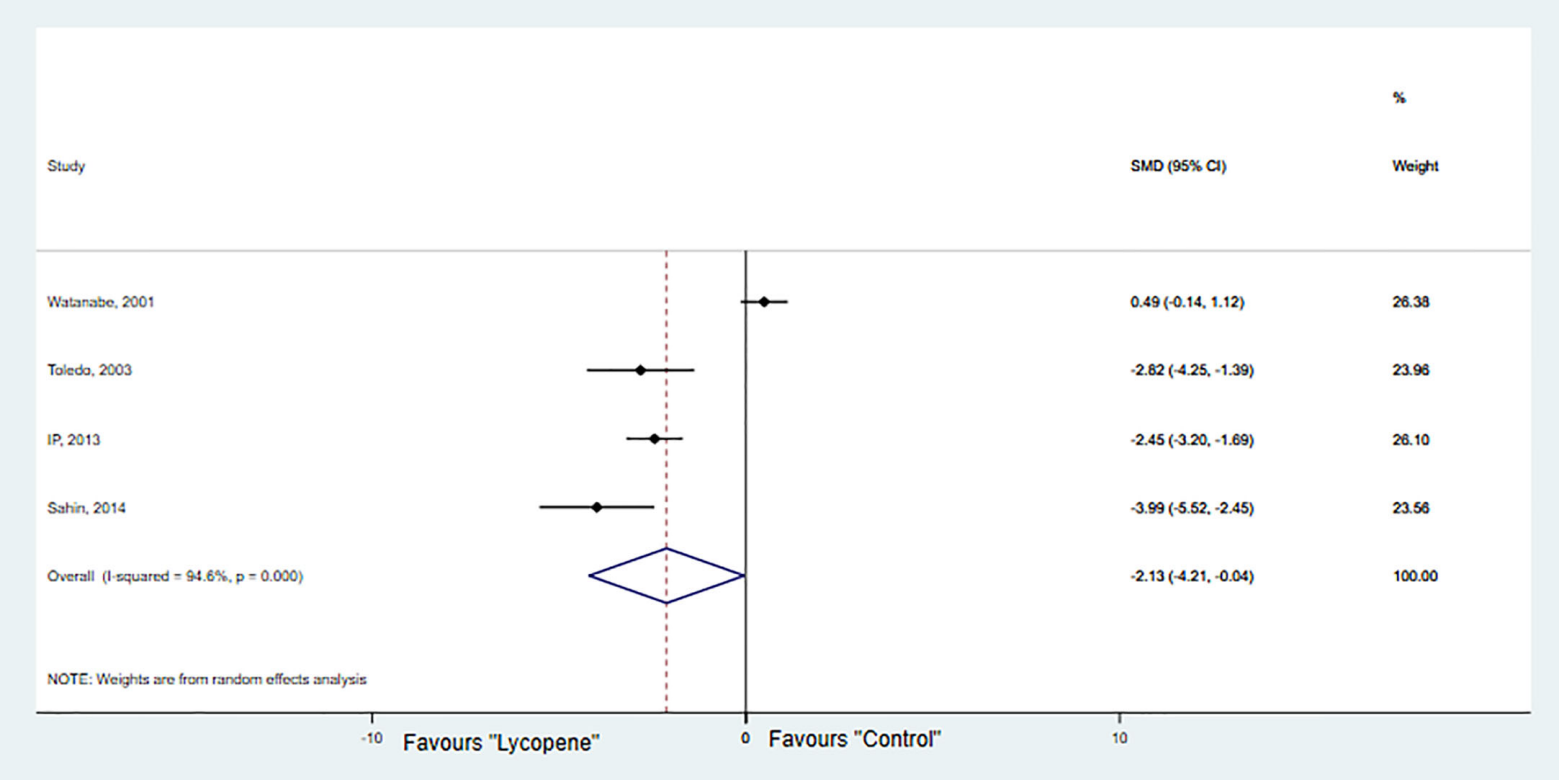
Forest plot of the effects of lycopene on tumor growth.

Four studies with 4 independent experiments were included to investigate the effect of lycopene treatment on HCC growth. The pooled estimate suggested a significant inhibition of HCC growth [SMD -2.13; 95% CI -4.20, -0.04 (*p*=0.04); I^2^ = 94.6%, *p*=0.00] (**Figure 4**). Due to the limited availability of data, it was not possible to conduct subgroup analyses.

### Publication Bias

**Figure 5:**
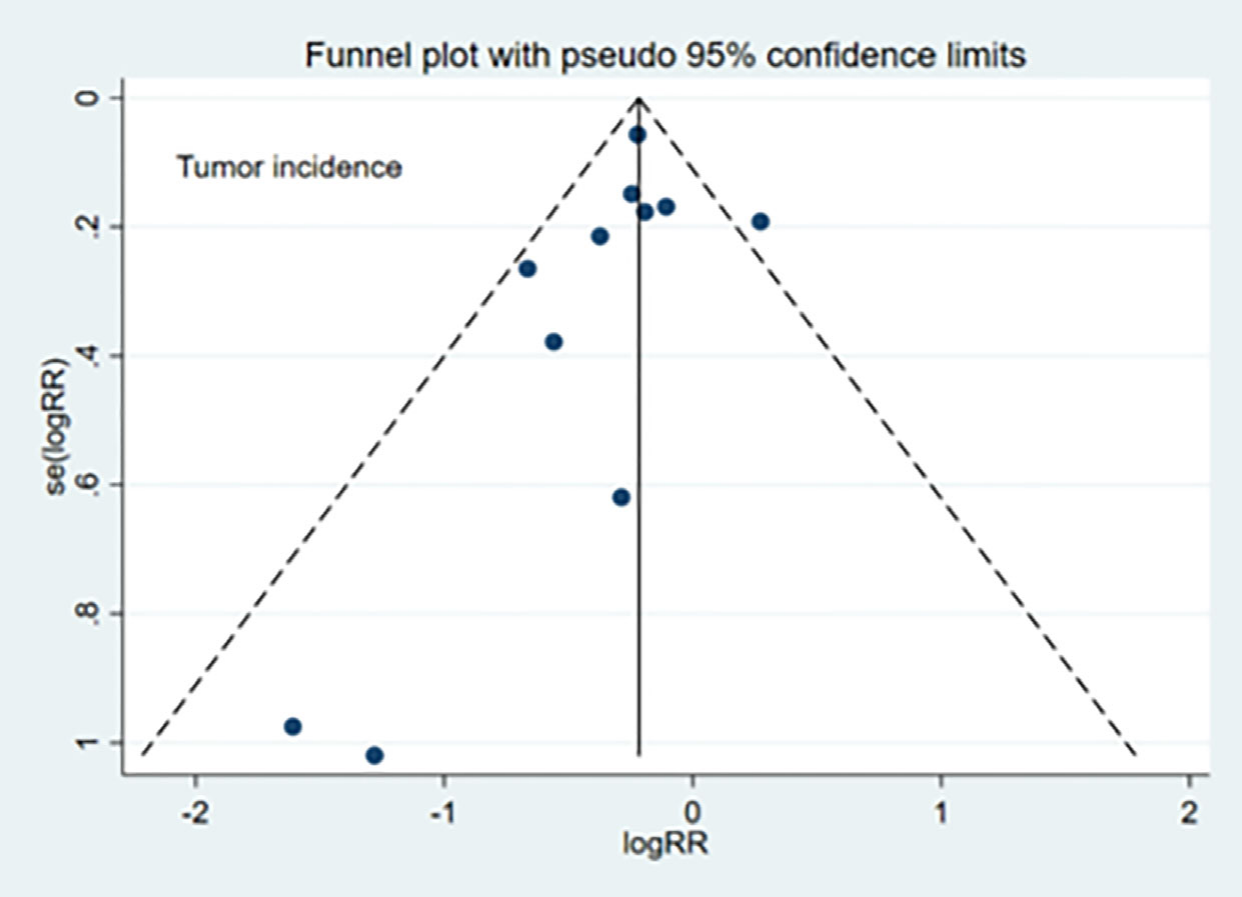
Funnel plots depicting publication bias.

The publication bias evaluation for the meta-analysis of tumor incidence (11 studies) is shown in **Figure 5**. No significant publication bias was observed for TI (Egger’s test: *p*=0.30), TN (Egger’s test: *p*=0.98), or TG (Egger’s test: *p*=0.26).

Sensitivity analysis showed that no studies substantially influenced the overall RR/SMD after removing one study at a time (**Table 3**).

**Table 3:** Sensitivity analysis to evaluate the effect of each individual study.

Outcome	Study omitted	RR/SMD	95%CI
Tumor incidence	Toledo et al., 2003	0.81	0.70,0.94
	Takahashi et al., 2010	0.78	0.67,0.92
	Takahashi et al., 2010	0.79	0.67,0.94
	Takahashi et al., 2010	0.78	0.72,0.86
	Wang et al., 2010	0.81	0.70,0.92
	Gupta et al., 2013c	0.82	0.72,0.94
	Ip et al., 2014	0.80	0.67,0.95
	Ip et al., 2014	0.79	0.64,0.96
	Sahin et al., 2014	0.81	0.69,0.95
	Aizawa et al., 2016	0.80	0.69,0.93
	Aizawa et al., 2016	0.80	0.70,0.93
	**Overall**	0.80	0.69,0.92
Tumor number	Toledo et al., 2003	-1.72	-3.08,-0.36
	Takahashi et al., 2010	-2.05	-3.42,-0.67
	Takahashi et al., 2010	-2.08	-3.43,-0.73
	Takahashi et al., 2010	-2.14	-3.41,-0.87
	Gupta et al., 2013c	-1.98	-3.41,-0.54
	Ip et al., 2013	-1.50	-2.79,-0.21
	Ip et al., 2014	-1.68	-3.08,-0.29
	Ip et al., 2014	-1.58	-2.76,-0.40
	Sahin et al., 2014	-1.79	-3.18,-0.40
	**Overall**	-1.83	-3.10,-0.57
Tumor growth	Watanabe et al., 2001	-2.89	-3.75,-2.04
	Toledo et al., 2003	-1.91	-4.44,0.62
	Ip et al., 2013	-2.05	-5.06,0.96
	Sahin et al., 2014	-1.55	-3.82,0.73
	**Overall**	-2.13	-4.21,-0.04

CI, confidence interval; RR, Risk Ratio; SMD, standardized mean difference.

## Discussion

This systematic review and meta-analysis was conducted to evaluate the effect of lycopene against animal models of HCC. We found that lycopene proved to generate a robust antitumor activity in animal models of HCC. Various mechanisms have been pointed out to explain the antitumor activity of lycopene against HCC development and progression. Although several *in vitro* and *in vivo* studies reported beneficial effects of lycopene against HCC, to the best of our knowledge, this is the first meta-analysis that assessed the anticancer activity of lycopene in animal models of HCC.

Lycopene has been shown to inhibit liver tumor initiation by diverse mechanisms including suppressing the expression of cytochrome p450 2E1 enzymes (involved in the activation of procarcinogens), scavenging oxygen free radicals or up-regulating the antioxidant defense system (Wang et al., 2010; Stice et al., 2015; Aizawa et al., 2016). The latter due to its ability to stimulate the nuclear factor E2-related factor 2 (Nrf2) that is known to be a crucial regulator of the cellular response to oxidative stress (Wang et al., 2010). Nrf2 has been shown to induce the expression of antioxidant or detoxifying enzymes such as superoxide dismutase (SOD), glutathione-S-transferase (GST), glutathione peroxidase (GPx), heme oxygenase-1 (HO-1), and catalase (Gupta et al., 2013b; Bhatia et al., 2018).

Moreover, several studies reported lycopene’s ability to inhibit HCC promotion and progression *via* altering the expression of genes involved in cell signaling, which are known to regulate cell proliferation and apoptosis (Gupta et al., 2013a; Ip et al., 2013; Sahin et al., 2014; Bhatia et al., 2015; Aizawa et al., 2016; Gupta et al., 2016). Lycopene has been shown to inhibit the proliferation of tumor cells *via* inducing cell cycle arrest (Teodoro et al., 2012; Takeshima et al., 2014). In this regard, lycopene prevents cell-cycle progression from G_1_ to S phase, mainly by inhibiting the expression of cyclin D and E that resulted in inactivation of cyclin-dependent kinase (CDK) 2 and 4 and associated decrease in the phosphorylation of retinoblastoma (Lurje et al., 2019) and inhibition of cell-cycle progression from G_1_ to S phase. Moreover, lycopene increases the expression of CDK inhibitors including p21 and p27, as well as the tumor suppressor gene p53 (Gupta et al., 2016).

Furthermore, studies have reported the ability of lycopene to induce apoptosis by down-regulating the expression of anti-apoptotic proteins, including Bcl-2, BclXL, and surviving and by up-regulating the expression of the pro-apoptotic proteins Bax, Bad, and Bim and associated activation of caspases 8, 9, and 3 (Gupta et al., 2013a; Jeong et al., 2019). In addition, lycopene has been shown to exert an anti-inflammatory effect to inhibit HCC development and progression (Wang et al., 2010; Ip et al., 2013; Ip et al., 2014; Stice et al., 2015; Bhatia et al., 2018). This is largely mediated through inhibition of the oncogenic transcriptional nuclear factor-kappa B (NF-κB) activation and downstream cascades (Ip et al., 2013; Bhatia et al., 2018). Hence, lycopene inhibits NF-κB induced expression of inflammatory cytokines including tumor necrosis factor alpha (TNFα) and interleukin (IL) 6, that affect the survival and proliferation of premalignant cells (Vendrell et al., 2015; Todoric et al., 2016). In addition, lycopene halts the activation of signal transducer and activator of transcription (STAT) 3, a transcription factor that is normally activated by IL6 and found to be important for HCC development (He and Karin, 2011; Fan et al., 2013).

The strength of this review is the use of evidence generated from controlled randomized experiments and prospectively collected data. However, our review also has some limitations that should be considered during interpretation of findings. *First*, the use of different species of animals made controlling the contribution of inter-species differences. We addressed this through sub-group analysis for each species. But given the overall small number of experiments, such sub-group analysis may not yield strong evidence. *Second*, the overall small number of animals in the study and its corresponding small number of experiments should be considered.

## Conclusion

The current meta-analysis suggests that lycopene has a potential to prevent HCC development and progression through its effect on the incidence, number and growth of HCC. This occurs as a result of antioxidant, anti-inflammatory, antiproliferative, and apoptosis inducing actions of lycopene in animal models of HCC. However, further controlled studies are required to support these mechanisms.

## Author Contributions

AM, BH, KM, and BM were involved in the conception, design, analysis, interpretation, and manuscript writing. AT, JA, and MS were involved in the design, analysis, and critically reviewing the manuscript. All authors contributed to the article and approved the submitted version.

## Conflict of Interest

The authors declare that the research was conducted in the absence of any commercial or financial relationships that could be construed as a potential conflict of interest.

## Abbreviations

CDK, Cyclin-dependent kinase; GPx, Glutathione peroxidase; GST, Glutathione-S-transferase; HO-1, Heme oxygenase-1; IL, Interleukin; NF-κb, Nuclear factor-kappa B; Nrf2, Nuclear factor E2-related factor 2; SMD, Standardized mean difference; SOD, Superoxide dismutase; STAT, Signal transducer and activator of transcription; TI, Tumor incidence; TN, Tumor number; TNFα, Tumor necrosis factor alpha; TS, Tumor size; TV, Tumor volume; VEGFR-2, Vascular endothelial growth factor-2.
