# Supplementary material for: Anti-Cancer Effects of Lycopene in Animal Models of Hepatocellular Carcinoma: A Systematic Review and Meta-Analysis
Source: Front Pharmacol. 2020 Aug 21;11:1306. doi: 10.3389/fphar.2020.01306 (PMC7475703; doi:10.3389/fphar.2020.01306)
Supplement: Supplementary file 2 [file Table_2.doc]

**Table S2. PubMed search strategy.**

| **Search** | **Query** | **Items found** |
| --- | --- | --- |
| #1 | Search ((((liver OR hepat*[tiab]) AND (carcino*[tiab])) OR (hepatoma*[tiab] OR (hepatocell*[tiab])) AND cancer*[tiab]) OR (hepatocarcinom*[tiab] OR AH 109a OR AH109a OR AH 130 OR AH130 OR AH 272 OR AH272 OR AH 66 OR AH66 OR HepG2 OR Hep G2 OR hcc) | 244370 |
| #2 | Search ((carotenoids*[tiab]) OR tomato*[tiab]) OR lycopene*[tiab] | 40032 |
| #3 | Search ((((mice) OR mouse) OR rat) OR rats) OR animal* | 7007490 |
| #4 | Search (((((((liver OR hepat*[tiab]) AND (carcino*[tiab])) OR (hepatoma*[tiab] OR (hepatocell*[tiab])) AND cancer*[tiab]) OR (hepatocarcinom*[tiab] OR AH 109a OR AH109a OR AH 130 OR AH130 OR AH 272 OR AH272 OR AH 66 OR AH66 OR HepG2 OR Hep G2 OR hcc))) AND (((carotenoids*[tiab]) OR tomato*[tiab]) OR lycopene*[tiab])) AND (((((mice) OR mouse) OR rat) OR rats) OR animal*) | 97 |
