# Supplementary material for: Anti-Cancer Effects of Lycopene in Animal Models of Hepatocellular Carcinoma: A Systematic Review and Meta-Analysis
Source: Front Pharmacol. 2020 Aug 21;11:1306. doi: 10.3389/fphar.2020.01306 (PMC7475703; doi:10.3389/fphar.2020.01306)
Supplement: Supplementary file 3 [file Table_3.doc]

Table S3. Quality assessment of included studies.

| **Author & year** | Publication in peer-reviewed journal | Statement of control of temperature | Randomization to treatment or control | Allocation concealment | Blinded assessment of outcome | Use of suitable animal model of HCC | Sample size calculation | Avoidance of anesthetics with marked intrinsic properties | Statement of compliance with regulatory requirements | Statement of potential conflicts of interest | **Total** |
| --- | --- | --- | --- | --- | --- | --- | --- | --- | --- | --- | --- |
| Watanabe, 2001 | ✓ | ✓ | - | - | - | ✓ | - | ✓ | - | - | 4 |
| Toledo, 2003 | ✓ | ✓ | - | - | - | ✓ | - | ✓ | ✓ | - | 5 |
| Takahashi, 2010 | ✓ | ✓ | - | - | - | ✓ | - | ✓ | ✓ | - | 5 |
| Wang, 2010 | ✓ | - | ✓ | - | ✓ | ✓ | - | ✓ | ✓ | ✓ | 7 |
| Gupta, 2013a | ✓ | - | ✓ | - | - | ✓ | - | ✓ | ✓ | - | 5 |
| Gupta, 2013b | ✓ | ✓ | ✓ | - | - | ✓ | - | ✓ | ✓ | ✓ | 7 |
| Gupta, 2013c | ✓ | ✓ | ✓ | - | - | ✓ | - | ✓ | ✓ | ✓ | 7 |
| IP, 2013 | ✓ |  | ✓ |  | ✓ | ✓ | - | ✓ | ✓ | - | 6 |
| IP, 2014 | ✓ | - | ✓ | - | ✓ | ✓ | - | ✓ | ✓ | - | 6 |
| Sahin, 2014 | ✓ | ✓ | - | - | - | ✓ | ✓ | ✓ | ✓ | - | 6 |
| Stice, 2015 | ✓ | - | ✓ | - | ✓ | ✓ | - | ✓ | ✓ | - | 6 |
| Bhatia, 2015 | ✓ | ✓ | ✓ | - | - | ✓ | - | ✓ | ✓ | ✓ | 7 |
| Aizawa, 2016 | ✓ | - | ✓ | - | ✓ | ✓ | - | ✓ | ✓ | ✓ | 7 |
| Gupta, 2016 | ✓ | ✓ | ✓ | - | - | ✓ | - | ✓ | ✓ | ✓ | 7 |
| Bhatia, 2018 | ✓ | ✓ | ✓ | - | - | ✓ | - | ✓ | ✓ | ✓ | 7 |
